# Biological Control of Three Major Cucumber and Pepper Pests: Whiteflies, Thrips, and Spider Mites, in High Plastic Tunnels Using Two Local Phytoseiid Mites

**DOI:** 10.3390/plants13060889

**Published:** 2024-03-20

**Authors:** Yusuf Abou Jawdah, Nour Ezzeddine, Aya Fardoun, Samer Kharroubi, Hana Sobh, Hagop S. Atamian, Margaret Skinner, Bruce Parker

**Affiliations:** 1 Department of Agriculture, Faculty of Agricultural and Food Sciences, American University of Beirut, Beirut P.O. Box 11-0236, Lebanon; nie08@mail.aub.edu (N.E.); af103@aub.edu.lb (A.F.); hs05@aub.edu.lb (H.S.); 2 Department of Nutrition and Food Sciences, Faculty of Agriculture and Food Sciences, American University of Beirut, Beirut P.O. Box 11-0236, Lebanon; sk157@aub.edu.lb; 3 Biological Sciences Program, Schmid College of Science and Technology, Chapman University, Orange, CA 92866, USA; atamian@chapman.edu; 4 Department of Plant and Soil Science, College of Agriculture and Life Sciences, University of Vermont, Burlington, VT 05405, USA; margaret.skinner@uvm.edu (M.S.); bruce.parker@uvm.edu (B.P.)

**Keywords:** food security, bio-based integrated pest management, two-spotted spider mites, whiteflies, thrips, aphids, *Amblyseius swirskii*, *Phytoseiulus persimilis*, biocontrol, greenhouse production

## Abstract

To enhance food security, food safety, and environmental health, a bio-based integrated pest management (BIPM) strategy was evaluated at two coastal locations in Lebanon as an alternative to toxic pesticide sprays in commercial high-arched plastic tunnels common in many countries. The evaluation occurred during two cucumber and pepper cropping seasons: spring and fall. At each site, two commercial tunnels were used; farmers’ conventional practices were applied in one tunnel, while the BIPM approach was followed in the second tunnel. In the farmers’ practices, a total of 14 sprays of insecticide/acaricide mixtures were applied during the spring growing season, and 6 sprays were applied during the fall. In the BIPM tunnels, hotspot releases of local strains of *Amblyseius swirskii* and *Phytoseiulus persimilis* were applied. By the end of the spring season, the number of whitefly nymphs (WFNs)/leaf and thrips/leaf in the pesticide treatment were 4.8 and 0.06, respectively, compared to 0.1 and 0.33, respectively, in the BIPM treatment. Similarly, at the end of the fall season, the WFNs reached 19.7/leaf in the pesticide control as compared to 1.2/leaf in the BIPM treatment, proving the efficacy of *A. swirskii*. Farmers using conventional acaricides during both cropping seasons failed to control *Tetranychus urticae*, the two-spotted spider mite (TSSM). However, hotspot releases of *P. persimilis* were successful in controlling TSSM. By the end of June, the number of TSSMs reached 7.8/leaf in the BIPM treatment compared to 53/leaf in the pesticide treatment. Likewise, in December, TSSM numbers reached 9/leaf in the BIPM treatment compared to 40/leaf in the pesticide treatment. Preliminary observations of pepper showed that both predatory mites (*A. swirskii* and *P. persimilis*) gave similar or better efficacy against the three pests. The two local predatory phytoseiid mites seem to be effective in controlling these three major pests and to be adapted to local environmental conditions. A rate of increase of 0.86 was observed for *P. persimilis* and 0.22 for *A. swirskii*, in June, when maximum temperatures were close to 40 °C. This also shows a compatibility between the two predators. In conclusion, our BIPM approach was efficient under a Mediterranean climate in arched plastic tunnels with relatively poor aeration.

## 1. Introduction

Food security and food safety have become a global priority, especially after the COVID-19 pandemic. The need to meet the demand for food from an ever-increasing world population is putting enormous stress on the limited natural resources to increase productivity per unit area and improve water use efficiency. Over the next 20 years, crop production will have to increase by 70% to meet the rising global population and accommodate changing diets in developing countries [1,2]. Among the productivity-enhancing technologies, protected cultivation has tremendous potential over open-field cultivation for achieving higher production per unit area [3]. It can provide high-quality products all year round with efficient use of resources, such as water, fertilizers, pesticides, and manual labor [4]. Crop production is practiced worldwide in greenhouses (GHs), including high plastic tunnels, as a tool to achieve food security, with an estimated global GH vegetable area of 496,800 hectares (ha) [5,6]. Cucumber (*Cucumis sativus* L.), belonging to the family Cucurbitaceae, is one of the most widely grown vegetable crops throughout the world [7], with a global production of 93 million tons of cucumbers and gherkins [8]. Sweet pepper (*Capsicum annum* L.), also known as bell pepper, belongs to the Solanaceae family and is the world’s second most important vegetable after tomato [9]. Globally, a total of 4 million tons of peppers were produced in 2021 [8]. 

While the microclimate in the GH provides optimal conditions for plant growth, it also creates a favorable environment for the proliferation of plant pests, which may cause severe yield losses if no appropriate pest management strategies are implemented. Among the various arthropod pests known to cause damage to vegetables, whiteflies (*Bemisia tabaci* Gennadius and *Trialeurodes vaporariorum* Westwood (Hemiptera, Aleyrodidae)), thrips (*Thrips tabaci* Lindeman and *Frankliniella occidentalis* Pergande (Thysanoptera: Thripidae)), aphids (*Aphis gossypi* Glover and *Myzus persicae* Sulzer (Hemiptera: Aphididae)), and the two-spotted spider mite (TSSM) *Tetranychus urticae* Koch (Acari, Tetranychidae) are the primary and most devastating arthropod pests affecting cucumber and tomato plants [10,11,12]. The sweet potato whitefly, *B. tabaci*, exhibits a broad host range, infecting over 1000 plant species, with biotypes MEAM1 and MED causing the most severe damage [13]. This whitefly species is a vector for more than 300 plant viruses, including geminiviruses, carlaviruses, nepoviruses, potyviruses, and closteroviruses [14,15,16]. *F. occidentalis* is a highly polyphagous species, targeting at least 250 plant species across more than 65 families [17]. It transmits viruses such as the *Tomato spotted wilt virus* (TSWV) and *Impatiens necrotic spot virus* (INSV) [18,19]. The two-spotted spider mite (TSSM), *T. urticae*, also has a wide host range and is a major pest of many crops, including cotton, ornamentals, strawberries, watermelons, cucumbers, peppers, tomatoes, and others [6]. 

Plant-infecting viruses transmitted by these polyphagous pests result in significant economic losses for the agriculture industry [20]. To address the issue of virus transmission, efficient pest control measures are essential. Historically, farmers have heavily relied on repeated pesticide applications to manage these pests. However, due to their phytophagous behavior, coupled with the rapid reproductive rates and short life cycles of these pests, resistance to numerous insecticides and acaricides has developed swiftly [21]. Another contributing factor to the rapid emergence of resistance in these pests is the limited scope of commercially employed insecticides. Specifically, most insecticides that are currently on the market target only six receptors, channels, or enzymes within the central nervous system and muscle physiology of insects [22]. Consequently, many destructive pest species have developed substantial resistance to these select classes of insecticides and acaricides. This resistance may arise through mechanisms such as overexpression of metabolic enzymes, facilitating rapid detoxification of the chemicals, or the selection of rare mutant alleles that confer target-site resistance [23]. Thus, the emergence of arthropod pest resistance against commonly applied insecticides and acaricides remains a serious issue in agriculture [24,25]. To exasperate the problem, some effective chemical ingredients have been restricted or phased out due to regulatory constraints. As a result, farmers have fewer options for pest control, leading to faster degradation of the efficacy of the remaining chemical ingredients. Moreover, in the absence of reliable alternatives, farmers resort to more frequent pesticide sprays, which negatively impact human health and the environment. Pesticide residues pose significant health risks to greenhouse workers and consumers. Altogether, these activities result in substantial financial losses for farmers due to crop damage, lower yields, and poor-quality products, affecting marketability. While swift solutions are necessary, developing new synthetic pesticides is not the answer. This is because the process of creating new active ingredients is complex and time-consuming, and regulatory hurdles delay their availability, as establishing the safety of new chemicals requires extensive testing [26].

In Lebanon, cucumber and pepper production in high-arched plastic tunnels relies heavily on the use of pesticides to control whiteflies, thrips, and spider mites. Thus, alternative approaches are needed to combat these devastating pests. Integrated pest management (IPM) has been recognized as an effective alternative strategy for managing pest populations. It involves integrating several environmentally friendly control practices [27], while minimizing the potential risk of developing insect/mite resistance to the pesticides used. This is achieved by alternating between cultural, biological, mechanical/physical, and chemical methods. The increased awareness among consumers regarding environmental and health issues, along with their demand for high-quality fruits and vegetables free from toxic pesticide residues, is among the main drivers for adopting bio-based integrated pest management (BIPM) in GH vegetable production. Biological control plays a crucial role in IPM programs and involves introducing natural enemies to control agricultural pests. Interest in biological control has grown over recent decades for several reasons [28]. It not only promotes the development of more sustainable farming practices but also effectively controls pests that have developed resistance to most synthetic chemicals [29]. Additionally, it addresses consumers’ demand for pesticide-free products [12]. Several commercially available predators and parasitoids are utilized in global commercial production. However, biological control agents may not always perform optimally across different geographical regions due to several factors, such as climatic and environmental conditions. This study was conducted in two coastal areas of Lebanon using local natural enemies. The results of this study may be of interest to farmers in similar regions/production conditions. *Amblyseius swirskii* Athias-Henriot (Acari, Phytoseiidae) and *Phytoseiulus persimilis* Evans (Acari, Phytoseiidae) are the most widely utilized predatory phytoseiid mites in GH settings. *A. swirskii* has garnered substantial interest as a biological control agent against thrips, whiteflies, and mites in GH and nursery crops [30]. *P. persimils*, on the other hand, is a specialist predatory mite employed for controlling spider mite species on vegetables and ornamentals [31]. Both species are commercially available and have been successfully used in various countries. It is believed that the origin of these two predatory mites is the Mediterranean region [32]. 

Biological control is often associated with climate-controlled greenhouses that offer optimal conditions [33]. In this study, we investigate the feasibility of implementing biological control within conventional high-arched plastic tunnels, where climate control is more challenging compared to modern commercial greenhouses. Specifically, we evaluated the efficacy and compatibility of two local strains of phytoseiid mites in developing a biological integrated pest management (BIPM) program. Our results demonstrate that biological control can indeed be successfully applied in plastic tunnels lacking precise climate control. By strategically deploying phytoseiid mites, we achieved effective pest reduction, maintaining the pest populations below the economic threshold levels. These findings have significant implications for sustainable cucumber and pepper production within high-arched plastic tunnels in Lebanon and many other Mediterranean regions with similar growing conditions. Importantly, it allows growers to minimize reliance on extensive pesticide applications, thereby mitigating health risks associated with the use of pesticides.

## 2. Results

### 2.1. Site I (Spring Crop)

#### 2.1.1. Plastic Tunnel Environmental Conditions

The average daily temperature during the experiment in these commercial plastic tunnels was 23.6 °C, with average maximum and minimum temperatures of 31 °C and 11.4 °C, respectively. The recorded maximum temperature reached 42.1 °C, while the minimum was 10 °C. The average daily temperatures varied between weeks, increasing as the season progressed. The temperature rose from 24.6 °C in week 2 to 26.51 °C in week 10. The relative humidity ranged from 51% to 90% (Figures S1 and S2).

#### 2.1.2. Control of Spider Mites on Cucumbers

**Figure 1:**
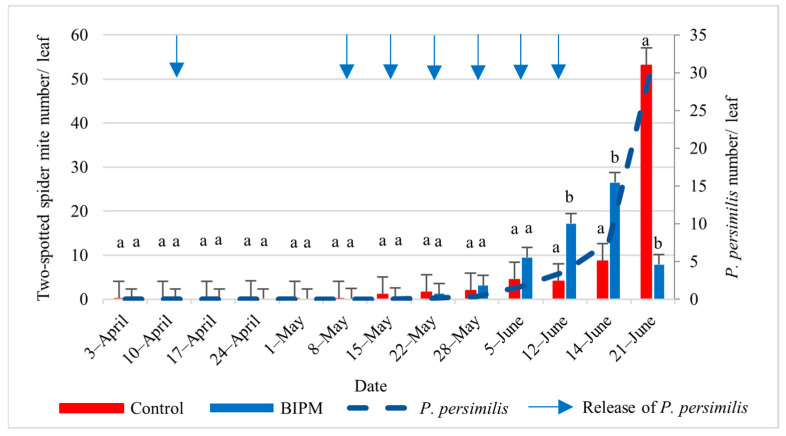
Average two-spotted spider mites (TSSM) and *P. persimilis* populations were recorded on cucumber leaves throughout the growing period in the control and BIPM tunnels, at Kfarmashoun. Different letters (a and b) indicate statistically significant differences in the pest numbers when comparisons are made between the two treatments (Control vs. BIPM) within the same week according to the Bonferroni post hoc test. Vertical blue arrows represent dates of natural enemy releases.

In the pesticide control tunnel, where the farmers applied 14 sprays of pesticide mixtures (Table S1), the TSSM population was maintained below the economic threshold (ET) level of 4 TSSM/cucumber leaf during the spring (Figure 1). However, with the increase in temperature during May–June, the TSSM population started to rise and exceeded the threshold level on June 5 (4.38 TSSM/leaf). Subsequently, the population increased to an average of 53 TSSM/leaf on June 21, experiencing a surge of 6.13 times during the last week (June 14–21), resulting in an intrinsic rate of increase of 0.69 from June 5 to June 21. It is noteworthy that, during June, the farmer employed three consecutive sprays of pesticide mixtures containing the acaricides tolfenpyrad, abamectin, or abamectin + pyridaben. Unfortunately, these sprays proved ineffective against TSSM. Consequently, the farmer decided to stop the production cycle on June 21, two weeks earlier than planned.

In the BIPM tunnel, *P. persimilis* was released to suppress the TSSM population on cucumbers and peppers (Table S2). During the early stage of the growing cycle, 2500 *P. persimilis* adults were released in the plastic tunnel to protect against the overwintering stages of spider mites on pepper plants. In the first period of the growing season (April to mid-May), both TSSM and *Phytoseiulus* populations were negligible, and the released predatory mite was not established in the plastic tunnel due to the rapid consumption of its prey (Table S1). 

Starting from mid-May, the TSSM infestation became more apparent and was concentrated on one side of the plastic tunnel, covering an estimated area of 250 m^2^ out of 450 m^2^ (infested area + buffer zone). Therefore, hotspot treatments of *P. persimilis* were applied at a rate of 4 predatory mites/m^2^ at low infestation levels of spider mites until May 22, increasing to 20 predatory mites/m^2^ on May 29. The TSSM population exceeded the ET of 4 TSSM/leaf from June 5, reaching a peak of 26.44 TSSM/leaf on June 14. However, it rapidly dropped within one week, by June 21, to reach 8 TSSM/leaf, compared to 53/leaf in the pesticide tunnel. 

This decline in the TSSM population was correlated with the establishment and increase in the number of *P. persimilis*. The ratio of *P. persimilis* to TSSM reached 1:5.71 on June 5, with the TSSM population continuing to increase but was accompanied by a simultaneous increase in the *P. persimilis* to reach a ratio of 1:4.8 (*P. persimilis*/TSSM) on June 11 and 1:3.46 ten days later. During this period, from June 12 to June 21, the TSSM population increased 13 times in the pesticide control treatment (from 4.06 to 53.05 TSSM/leaf) despite application of two pesticide sprays, while it dropped by 54% (from 17.2 to 7.88 TSSM/leaf) in the BIPM treatment. This drop was correlated with an 8.7 times increase in the *P. persimilis* population (from 3.6 to 31.3/leaf), demonstrating the high efficacy of the local strain of *Phytoseiulus* at the high temperatures prevailing in June (maximum temperature fluctuating between 32 °C and 39 °C, with an average temperature 25.9 °C and 75% RH).

There was no significant difference in the number of TSSMs between the two plastic tunnels (*p* = 0.352), but a significant difference was observed in the number of TSSMs over weeks x treatments (*p* = 0.000).

#### 2.1.3. Control of Spider Mites on Peppers

**Figure 2:**
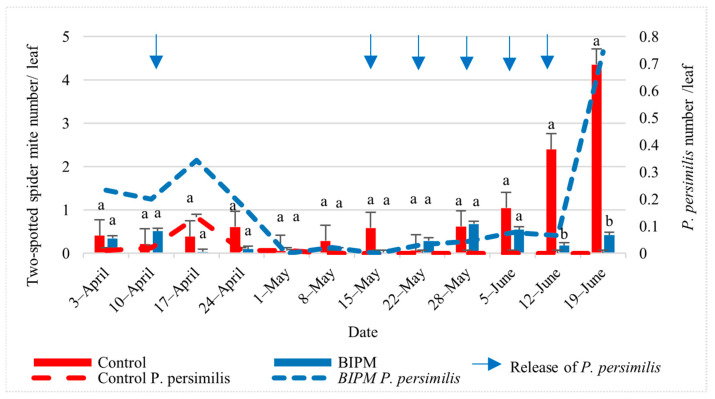
Average two-spotted spider mites (TSSM) and *P. persimilis* populations were recorded on pepper leaves throughout the growing period in the control and BIPM tunnels, at Kfarmashoun. Different letters (a and b) indicate statistically significant differences in the pest numbers when comparisons are made between the two treatments (Control vs. BIPM) within the same week according to the Bonferroni post hoc test. The red dotted line represents control *P. persimilis* (showing migration into the tunnel before releasing the *P. persimilis* as treatment) and the blue dotted line represents the treatment (*P. persimilis* was released as part of the BIPM treatment). Vertical blue arrows represent dates of natural enemy releases.

On peppers (Figure 2), residual TSSM populations on plants maintained from the previous growing season were observed in the pesticide tunnel from the beginning of April. However, pesticide sprays kept it under the ET of two TSSM/leaf [34] until June 5. The population then increased rapidly (r = 0.2), from 1.04 to 4.39/leaf (4.2 times) within 14 days, between June 5 and June 19, exceeding the ET with two peaks of 2.4 and 4.35 mite/leaf, on the 12th and 19th of June despite multiple pesticide sprays. The active ingredients used did not seem to prevent the population outbreak. In the BIPM tunnel, the releases of *P. persimilis* kept the TSSM population on peppers below the ET throughout the entire growing season. Within 14 days, between June 5 and June 19, the population of *P. persimilis* increased, and the TSSM population was reduced by 22% compared to a TSSM population increase of 4.3 times in the pesticide control. The ratio of *P. persimilis*/TSSM was 1:15.25 on May 29, 1:7 on June 5, and 1:0.57 on June 19, with a peak of 0.74 Phytoseiulus/leaf. There was a significant difference in the number of mites between the two pepper treatments (*p* = 0.000) and in the number of spider mites in the weeks x treatments (*p* = 0.000).

#### 2.1.4. Control of Whiteflies on Cucumber

**Figure 3:**
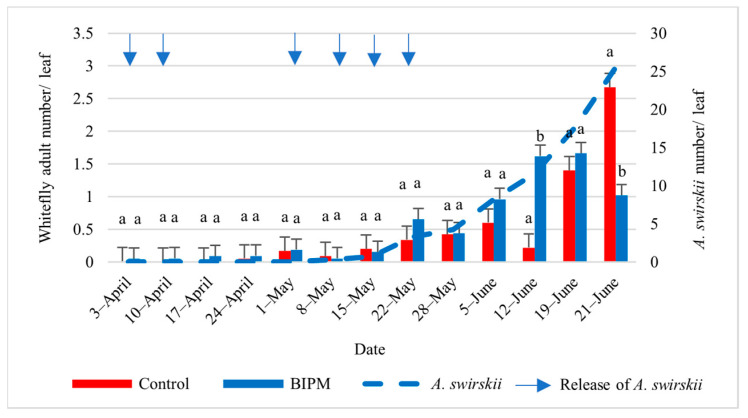
Average whitefly adults and *A. swirskii* populations were recorded on cucumber leaves throughout the growing period in the control and BIPM tunnels, at Kfarmashoun. Different letters (a and b) indicate statistically significant differences in the pest numbers when comparisons are made between the two treatments (Control vs. BIPM) within the same week according to the Bonferroni post hoc test. Vertical blue arrows represent dates of natural enemy releases.

**Figure 4:**
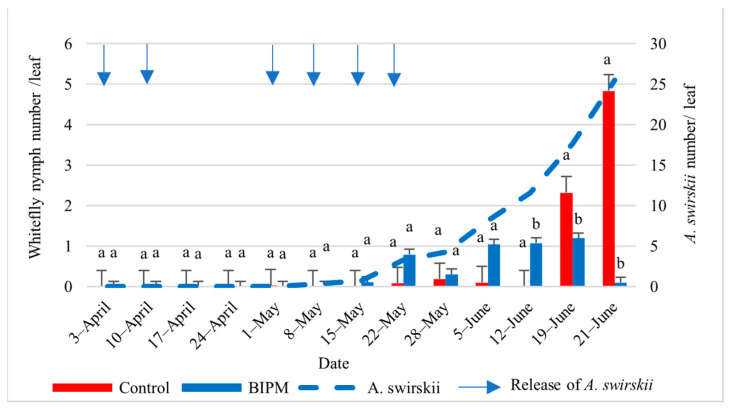
Average whitefly nymphs and *A. swirskii* populations were recorded on cucumber leaves throughout the growing period in the control and BIPM tunnels, at Kfarmashoun. Different letters (a and b) indicate statistically significant differences in the pest numbers when comparisons are made between the two treatments (Control vs BIPM) within the same week according to the Bonferroni post hoc test. Vertical blue arrows represent dates of natural enemy releases.

In the pesticide control tunnel, with 14 pesticide sprays, the farmer maintained the whitefly nymph (WFN) and whitefly adult (WFA) numbers on leaves below the ET of 4.6 WFN/leaf [5] throughout the growing season. The first clear WFA and WFN counts were recorded from early May, and the WF population numbers remained low but peaked on June 19 and 21 with 2.32 and 4.83 WFN/leaf and 1.4 and 2.67 WFA/leaf, respectively. Similar control efficacy was observed in the BIPM GH, with seven releases of SW. The whitefly adult and nymph populations were also kept below the ET [35] for the entire season. The maximum number of WF reached a peak of 1.67 WFA/Leaf on June 12 and 19 and dropped to 1 WFA on June 21, while the nymph population was maintained at about 1 WFN/L or less from the beginning of May to June 19, dropping to 0.1 WFN/Leaf on June 21. The drop in the WFN population was correlated with a steady increase in the *A. swirskii* population, with the *A. swirskii* intrinsic rate of increase in June reaching 0.22, indicating that the local *A. swirskii* strain tolerates the hot weather conditions present in the plastic tunnel from Mid-May to June (Figure 3 and Figure 4).

#### 2.1.5. Control of Whiteflies on Pepper

**Figure 5:**
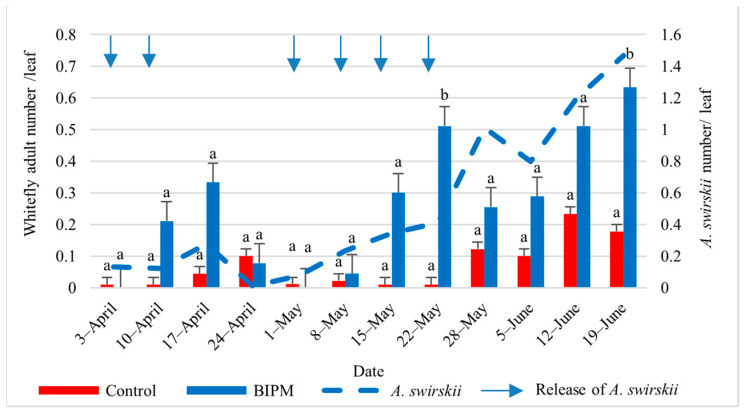
Average whitefly adults and *A. swirskii* populations were recorded on pepper leaves throughout the growing period in the control and BIPM tunnels, at Kfarmashoun. Different letters (a and b) indicate statistically significant differences in the pest numbers when comparisons are made between the two treatments (Control vs BIPM) within the same week according to the Bonferroni post hoc test. Vertical blue arrows represent dates of natural enemy releases.

**Figure 6:**
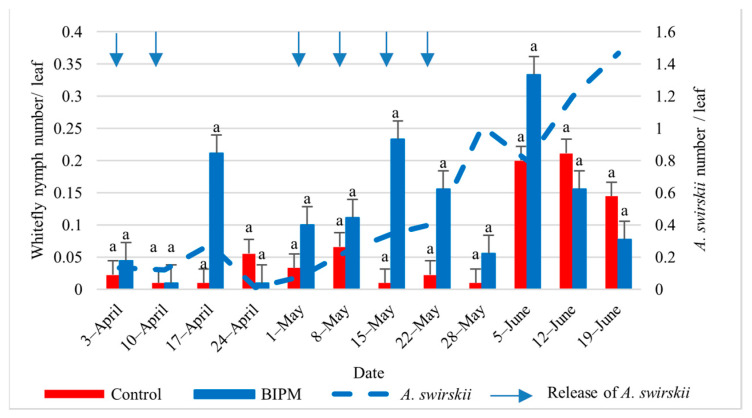
Average whitefly nymphs and *A. swirskii* populations were recorded on pepper leaves throughout the growing period in the control and BIPM tunnels, at Kfarmashoun. Different letters (a and b) indicate statistically significant differences in the pest numbers when comparisons are made between the two treatments (Control vs. BIPM) within the same week according to the Bonferroni post hoc test. Vertical blue arrows represent dates of natural enemy releases.

On peppers, both the pesticide control and BIPM treatments effectively suppressed the population of WF adults and nymphs considerably below their ETs. Unlike on cucumber, the counts of WF nymphs and adults observed in the first two weeks of the trial were not negligible, showing residual infestations from the previous season (Figure 5 and Figure 6).

The WF populations fluctuated during the season at levels considerably below the ETs, with a peak of 0.23 WFA and 0.21 WFN/leaf in the pesticide control tunnel on June 12 compared to 0.511 WFA and 0.15 WFN/leaf in the BIPM tunnel, with no significant difference between the two treatments (*p*WFA = 0.17 and *p*WFN = 0.6). The highest peak in the BIPM tunnel was registered at 0.63 WFA and 0.33 WFN/Leaf. The *A. swirskii* population increased continuously starting from early May, with an intrinsic rate of increase of 0.36 during May and June.

#### 2.1.6. Control of Thrips on Cucumber

**Figure 7:**
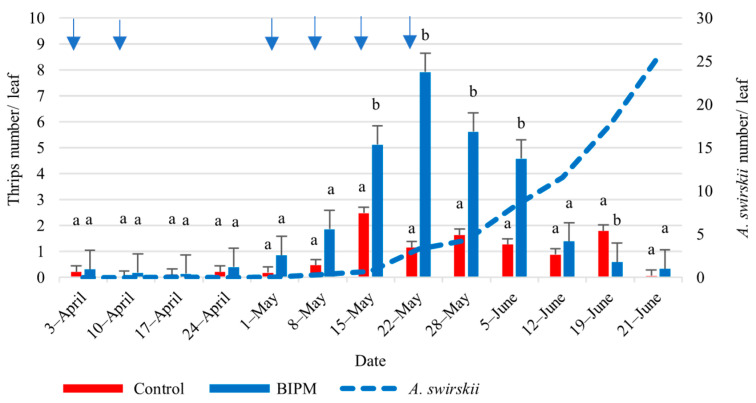
Average thrips and *A. swirskii* populations were recorded on cucumber leaves throughout the growing period in the control and BIPM tunnels, at Kfarmashoun. Different letters (a and b) indicate statistically significant differences in the pest numbers when comparisons are made between the two treatments (Control vs BIPM) within the same week according to the Bonferroni post hoc test. Vertical blue arrows represent dates of natural enemy releases.

At site I, in the pesticide tunnel, the population of thrips on cucumber leaves fluctuated at low levels, with a peak of 2.5 thrips/leaf on May 15 (Figure 7).

In the BIPM tunnel, the thrips population growth pattern resembled a bell-shaped curve, where nymphs started increasing from April 24 to reach a peak of 7.9 thrips/leaf on May 22, and then it decreased at a rapid rate to reach 0.33 thrips/leaf on June 21. Thus, the ET was slightly exceeded for a period of 3 weeks (weeks 7–9). The drop in the thrips population starting from May 22 was correlated with a continuous increase in the *A. swirskii* population until the end of the season at an intrinsic rate of increase of r = 0.22. The ratio of *A. swirskii*/thrips on May 15, a week before the population of larvae reached its peak, was 1:7. Then this ratio of *A. swirskii*/thrips increased to 1:2.4 on May 22 and further increased to reach 1:0.01 on June 21.

When the statistical analysis was run over the whole experimental period, no significant difference in thrips numbers was observed between the pesticide control and the BIPM treatments. However, a significant difference in the thrips population between the pesticide and the BIPM tunnels was recorded between May 15 and June 5 (*p*May15 = 0.00, *p*June5 = 0.00).

#### 2.1.7. Control of Thrips on Pepper

**Figure 8:**
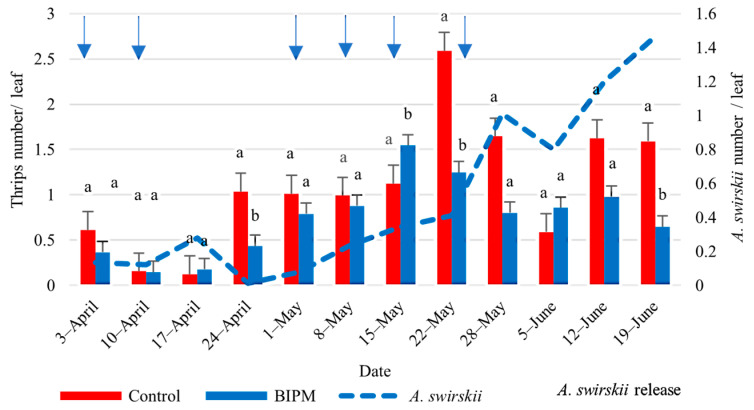
Average thrips and *A. swirskii* populations were recorded on pepper leaves throughout the growing period in the control and BIPM tunnels, at Kfarmashoun. Different letters (a and b) indicate statistically significant differences in the pest numbers when comparisons are made between the two treatments (Control vs. BIPM) within the same week according to the Bonferroni post hoc test. Vertical blue arrows represent dates of natural enemy releases.

In both the pesticide and control BIPM tunnels, the population of thrips adults and nymphs was maintained below the ET throughout the growing season. The population densities followed the same pattern as observed on cucumber, with a peak number of thrips (adults and nymphs) on May 22, reaching 2.58 thrips/leaf in the pesticide control tunnel, which was significantly higher than the thrips number (1.54 thrips/leaf) in the BIMP tunnel (*p* = 0.001) (Figure 8).

### 2.2. Site II (Fall Crop)

#### 2.2.1. Plastic Tunnel Environmental Conditions

The average temperature for the growing season was 25.26 °C, with great variations during the season; for example, it varied between 31.28 °C on week 2 and 19.08 °C on week 10. The average minimum and maximum temperatures were 17 and 39.5 °C with the highest temperature recorded at 54.23 °C on September 21. The relative humidity fluctuated between 51% and 90% (Figure S3).

#### 2.2.2. Control of Spider Mites on Cucumber

**Figure 9:**
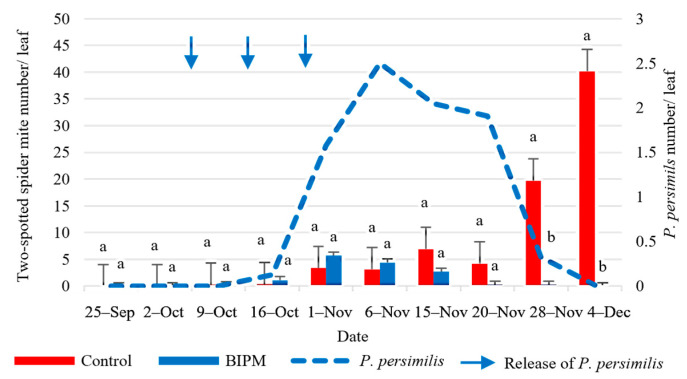
Average two-spotted spider mites (TSSM) and *P. persimilis* populations were recorded on cucumber leaves throughout the growing period in the control and BIPM tunnels, at Rmeileh. Different letters (a and b) indicate statistically significant differences in the pest numbers when comparisons are made between the two treatments (Control vs. BIPM) within the same week according to the Bonferroni post hoc test. Vertical blue arrows represent dates of natural enemy releases.

In the pesticide control tunnel, with six sprays of pesticide mixtures (Table S3), the farmer was able to maintain the TSSM population below the ET of 4 TSSM/leaf until November 15, where it exceeded the ET and reached 7 TSSM/leaf, and then it increased to reach 19.7 and 40 TSSM/leaf on November 28 and December 4, respectively (Figure 9). Note that the last pesticide spray was on November 20 and included only one specific acaricide. The intrinsic rate of increase between November 20 and December 4 was 0.6, a relatively high rate compared to the season with an average temperature of 17 °C, with a maximum temperature fluctuating between 20 °C and 50 °C and a minimum temperature ranging between 11.12 °C and 14.5 °C, during this period. In the BIPM tunnel, the TSSM population was suppressed below the ET all through the growing season, except for the first week of November where it reached a peak of 5.5 TSSM/leaf and dropped thereafter. During the period of October 16 to November 6, the intrinsic rate of increase in the population of *P. persimilis* was r = 0.125. On November 1, the population density of *P. persimilis* reached 1.5/leaf and increased to 2.5 predatory mites/leaf on November 6. The ratio of predator/prey was 1:1.76 on November 6, peaking at 1:1.32 one week later, but it dropped thereafter due to the diminishing number of prey. By the end of the season, the spider mite population in the BIPM tunnel was negligible, exhibiting a significantly lower count as compared to the control group, which reached 40 TSSM/leaf on December 4 (*p* = 0.000).

#### 2.2.3. Control of Whitefly on Cucumber

In the control tunnel, the farmer applied six insecticidal/acaricidal sprays throughout the growing period using three active ingredients per application (Table S3). The population of adult whiteflies (WFA) remained below one adult per leaf during the first six weeks of the experiment until the beginning of November. During this period, the farmer had already applied three sprays. Then, the WFA increased at a steady rate to reach a peak of 4.12 WFA/leaf by mid-December, despite the application of three pesticide sprays during this period. In conclusion, the six pesticide sprays maintained the whitefly adult population below the ET of 4.6 WFA/Leaf. However, the pesticidal sprays were not as efficacious against the nymphal stages (WFN), which exceeded the ET of 4.6 WFN/leaf by mid-November and continued increasing to reach 19.7 WFN/leaf by December 4. 

In the BIPM treatment (Table S4), *A. swirskii* was more efficacious than the pesticide sprays. Two releases of *A. swirskii* at a rate of 25 mites/m^2^ were performed on the 1st and 3rd weeks of the experiment (Sep 25 and Oct 9), provided good control and maintained whiteflies at low levels (<0.5 adult or nymph/leaf) till October 16. From November on, while the number of nymphs was increasing in the control and largely exceeded the ET, the number of nymphs in the BIPM was maintained below the ET by two additional releases of *A. swirskii* at a rate of 50 mites/m^2^ on November 1st and two weeks later. By the end of the experiment, the number of WFN was 1.2 in the BIPM treatment as compared to 19.7 in the pesticide control. Overall, there was a significant difference in the average number of whitefly adults and nymphs between the two treatments (*p*WFA = 0.014; *p*WFN = 0.000).

**Figure 10:**
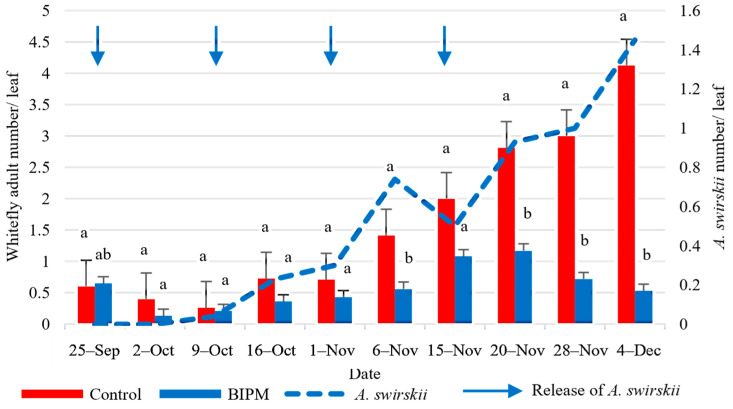
Average whitefly adults and *A. swirskii* populations were recorded on cucumber leaves throughout the growing period in the control and BIPM tunnels, at Rmeileh. Different letters (a and b) indicate statistically significant differences in the pest numbers when comparisons are made between the two treatments (Control vs BIPM) within the same week according to the Bonferroni post hoc test. Vertical blue arrows represent dates of natural enemy releases.

**Figure 11:**
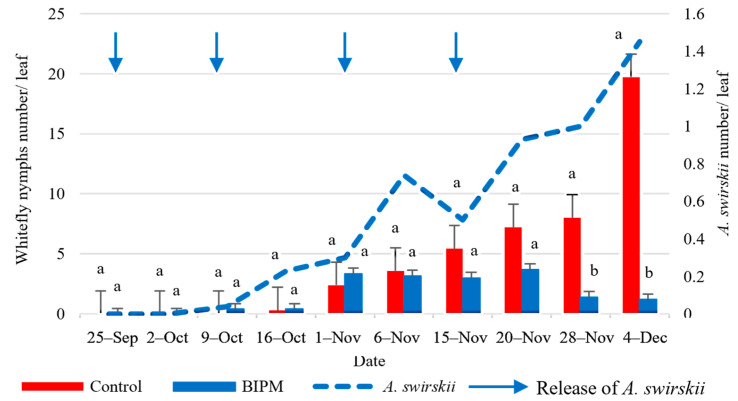
Average whitefly nymphs and *A. swirskii* populations were recorded on cucumber leaves throughout the growing period in the control and BIPM tunnels, at Rmeileh. Different letters (a and b) indicate statistically significant differences in the pest numbers when comparisons are made between the two treatments (Control vs BIPM) within the same week according to the Bonferroni post hoc test. Vertical blue arrows represent dates of natural enemy releases.

The ratio of *A. swirskii*/WFN reached 1:4.8 on November 1st and 1:2.12, two weeks later. The highest peak of *A. swirskii* was 1.45 predator/leaf, recorded on December 4. This increase in the *A. swirskii* population was correlated with a drop in the average whitefly population to a level of 0.53 adults and 1.2 nymphs per leaf by the end of the experiment, a reduction of 87.1 and 94%, respectively, compared to the pesticide control (Figure 10 and Figure 11).

#### 2.2.4. Control of Thrips

**Figure 12:**
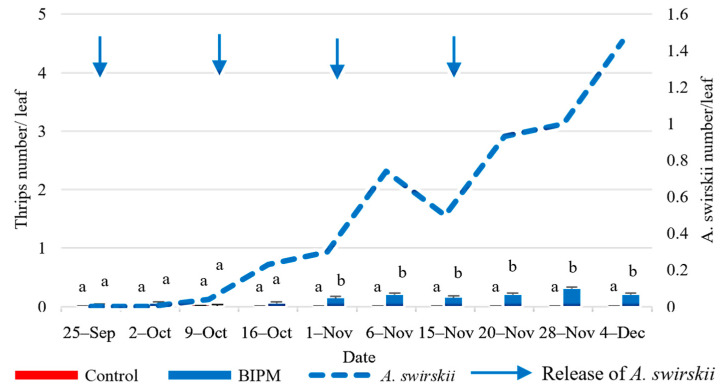
Average thrips and *A. swirskii* populations were recorded on cucumber leaves throughout the growing period in the control and BIPM tunnels, at Rmeileh. Different letters (a and b) indicate statistically significant differences in the pest numbers when comparisons are made between the two treatments (Control vs BIPM) within the same week according to the Bonferroni post hoc test. Vertical blue arrows represent dates of natural enemy releases.

In the pesticide tunnel, the thrips population was low (<0.2/Leaf) throughout the growing season (Figure 12). The applied insecticides were able to suppress the thrips population. The sprayed active ingredients were as follows: Abamectin, Acetamiprid, and Lambda-Cyhalothrin.

In the BIPM tunnel, the four *A. swirskii* releases, applied for whitefly management, also provided good control agaithe nst thrips population and maintained it below the ET throughout the growing period. After the last release of *A. swirskii*, on November 15, its population doubled within 2 weeks from 0.5 to 1 predator/leaf on Nov 28 (Figure 12), when the ratio of *A. swirskii*/thrips was 1:3.33. At this ratio, a 33% drop was recorded in thrips density within a week. A significant difference in the average number of thrips between the two treatments (*p* = 0.01) was observed from the first of November until the end of the season but remained below ET in both treatments. 

There was a significant difference in the average number of thrips between treatments (*p* = 0.000), weeks (*p* = 0.000), as well as treatment × week (*p* = 0.000).

## 3. Discussion

This study, conducted under commercial high-arched plastic tunnel growing conditions in the Mediterranean region, showed that BIPM may represent a viable alternative to pesticide sprays in the inexpensive high-arched plastic tunnels, which may not be considered as convenient for biocontrol as the more expensive double- or multiple-span GHs that are equipped with better climate control. This constitutes an essential component for improved food safety and food security. The trials covered the two main cropping seasons: the spring season from March to June and the fall season from September to December. Arthropod pest pressure is typically higher in the spring cropping season and at the beginning of the fall growing season. 

At both sites and cropping seasons, farmers faced difficulties in controlling TSSM using conventional acaricidal sprays. Determining the ET for TSSM on cucumber leaves was challenging, as no specific ET had been reported in the literature. The decision was based on the findings of a study [36] that showed a significant decrease in the economic yield of cucumber four weeks after plants were infested with four TSSM per 10 cm^2^ of leaf area at transplanting. Consequently, a conservative ET of 4 TSSM/leaf was adopted. This decision considered factors such as the relatively high leaf surface area of cucumber varieties grown in GHs, the high cash value of GH crops, allowing lower pest threshold levels as compared to field crops, and the high rate of TSSM increase during May and June. 

In the pesticide control tunnels, 14 sprays of pesticide mixtures were applied during the spring season and six during the fall season. Due to favorable environmental conditions in June, three sprays of pesticide mixtures, including acaricides, did not provide satisfactory control of the TSSM population. Because of the severity of the infestation, the farmer decided to stop production two weeks earlier than planned. The observed TSSM intrinsic rate of increase (r = 0.43) in June was higher than expected, suggesting that migration occurs from drying weeds near the tunnels into the production tunnels during this period. Therefore, starting from mid-May, it is recommended to monitor TSSM twice a week. In the BIPM tunnel, the local strain of *P. persimilis* was able to establish in the tunnel, and despite an occasional increase in the TSSM population above the ET for a short period, the predator population grew at a very fast rate and significantly reduced the TSSM population. 

Although TSSMs were present in both plastic tunnels, the new growth of cucumber leaves in the BIPM tunnel was greener and pest-free compared to the control tunnel (Figure 1). Additionally, plants in the BIPM tunnel were healthy and vigorous, while in the control tunnel, the plants displayed phytotoxicity symptoms and flower drop, likely resulting from the application of mixtures of insecticides/miticides during the hot weather in June (Figure 1). 

Similar observations were made at site II, where the TSSM population in the pesticide control tunnel exceeded the ET from mid-November until the first week of December, which marked the end of the experiment. Meanwhile, in the BIPM tunnel, the TSSM population was maintained below the threshold level throughout the entire growing season. In both growing seasons, the BIPM treatment demonstrated significant ecological benefits: in addition to the substantial reduction in pesticide use, it reduced the TSSM population that might otherwise migrate to nearby hosts upon crop removal. Moreover, the residual migration of TSSM leads to the widespread distribution of predatory mites. At site II, we observed *P. persimilis* in nearby open field beans about three months after the trial concluded. The predation activity of *P. persimilis* has been widely documented in both open fields and GH crops [28,32,37]. The efficacy of *P. persimilis* is correlated with the predator-prey release ratios and the timing of mite introduction [37]. High release ratios or early introductions may result in the rapid decline of spider mite populations. On the other hand, low release ratios or delayed introductions will lead to high spider mite infestations [38]. 

In the current study, the hotspot release of *P. persimilis* at a rate of 24 mites/m^2^ right after the first appearance of TSSM infestations was followed by subsequent releases attempting to reach a *P. persimilis*/TSSM ratio of 1:4. The hotspot release rate was double the rate (12 mites/m^2^) recommended at the beginning of infestation by international companies. This rate was required, during the most favorable period for TSSM development, as the predator was expected to take over TSSM. Additional releases may not be warranted unless this ratio drops, as *P. persimilis* is known for its cannibalism habit when it exhausts its feed supply [39]. Effectively, the population of *P. persimilis* dropped rapidly when the TSSM population became low. Our results are in general agreement with those of Opit [28], who reported keeping a consistent ratio of 1:4. *P. persimilis* to TSSM significantly reduced the populations of the TSSM on GH ivy geraniums. However, Yanar et al. [37], on GH-grown cucumbers, reported adequate control of TSSM at a moderately low predator/prey release ratio of 1:15, but better results were obtained at a ratio of 1:5. At the latter ratio, an initial increase in the TSSM population density above 4 TSSM/leaf was observed for a period of 3–4 weeks before the predatory mite was able to reduce TSSM numbers considerably. Similar results were observed in our trials, where a peak of TSSM was reached before the predator was able to establish and reduce the TSSM population significantly. The high ratio of predator to prey (1:4) required in this study may be explained by the exceptionally high intrinsic rate of growth of the TSSM population from mid-May onward, which was also correlated with the migration of TSSM into the tunnels from the surrounding environment. Under our experimental conditions, satisfactory control was obtained upon release of 9.6 and 40 *P. persimils*/m^2^, during the fall and spring seasons, respectively. 

At both locations and cropping seasons, the timely release of appropriate numbers of the local strain of *A. swirskii* provided highly effective control of whitefly nymphs and thrips throughout the growing season with minor exceptions. This predatory mite became well established on cucumber leaves, reaching 25.5 mites/leaf toward the end of June at site I. Similar results were reported by Messelink et al. [40], where 30 *A. swirskii*/cucumber leaf were observed. Several studies indicate that the viability and efficacy of *A. swirskii* increase when its feed source is based on a mixture of two or more prey species. For example, better control of Western flower thrips (*Frankliniella occidentalis*) and the GH whitefly (*Trialeurodes vaporariorum*) was achieved in a “1 predator-2 prey system”. Messelink et al. [40] and Calvo et al. [41] reported that *A. swirskii* shows promise in controlling whitefly nymphs, with a release rate of 25 and 100 predatory mites/m^2^. Additionally, Bolckmans et al. [30] reported that *A. swirskii* released at a rate of 200–240/m^2^ (80/plant) is adequate for controlling whitefly and thrips populations in cucumber GHs. Under our experimental conditions, efficient control was obtained with *A. swirskii* release rates of 150 and 267/m^2^ during the fall and spring crops, respectively. 

Our results are in agreement with previous reports regarding the ability of *A. swirskii* to suppress whiteflies on cucumber under field conditions [40,42] as well as its efficacy against *F. occidentalis* on cucumber and sweet pepper under laboratory and GH conditions [43,44,45].

Previous studies found that the introduction of multiple phytoseiid mites can mediate trophic interactions, such as competition for the same food source and inter- and intra-guild predation when the density of preferred prey is low [46]. Without an alternative and preferred food source, intraguild predation by *A. swirskii* mites on *P. persimilis* was observed [47]. However, effective pest control was achieved when the two mites were released in combination, each targeting a specific target: *A. swirskii* against *F. occidentalis* and *P. persimilis* against *T. urticae* [48]. The current work is in agreement with Lanzoni [49] and shows that introducing *A. swirskii* and *P. persimilis* based on the availability of their preferred prey allows for their simultaneous and efficient use. This is supported by the fact that both populations of *P. persimilis* and *A. swirskii* grew concurrently at a fast rate at both sites and in both growing seasons. At site I, during the growing period from June 5 to 21, both population densities of *P. persimilis* and *A. swirski* grew at intrinsic rates of increase of r = 0.86 and r = 0.22, respectively. Also at site II, during the period of October 16 to Nov 6, both population densities of *P. persimilis* and *A. swirskii* were increasing at a rate of 0.55 and 0.105, respectively, demonstrating compatibility between the two predators. 

The local *P. persimilis* and *A. swirskii* strains proved to be well-adapted to the environmental conditions prevailing in the high-arched plastic tunnels during May/June and September/October. Therefore, we have showed that BIPM can be successfully implemented even in high plastic tunnels with relatively poor aeration.

The promising preliminary results obtained on pepper, the second most important GH vegetable crop, suggest that the concurrent releases of *P. persimilis* and *A. swirskii* may be as effective or even more effective against the three reported pest species than observed on cucumber.

A well-planned BIPM strategy should consider all potential pests that may infest the crop and not only the three major pests mentioned in this study. Due to intensive pesticide use against thrips and whiteflies, many pests like aphids and leaf miners were not considered major pests. However, when the use of pesticides is stopped or reduced, they may emerge as major pests, and appropriate control measures, BIPM compatible, should be applied. 

The use of natural enemies is a safe approach for farmers, consumers, and the environment. However, their application cost should be evaluated under local conditions.

## 4. Materials and Methods

### 4.1. Biocontrol Agents

The *P. persimilis* colony was collected from bean plants, *Phaseolus vulgaris* L. (Fabaceae), grown in conventional high plastic tunnels, in Jiyeh, South Lebanon (33°39′56.51″ N 35°25′36.46″ E). It was reared at the American University of Beirut (AUB) greenhouses on bean plants infested with *T. urticae*.

The *A. swirskii* colony was collected from castor bean plants, *Ricinus communis* L. (Euphorbiaceae) from Batroun, North Lebanon (34°15′0″ N 35°39′0″ E). *A. swirskii* was reared at the AUB laboratory in controlled environment growth cabinets using a specially developed rearing method. 

To confirm the proper identification of both predators, in addition to morphological characterization, molecular tools based on partial sequencing of the ITS ribosomal DNA region were conducted as reported by [49]. This may be required for use registration by the governmental authorities.

### 4.2. Site Selection and Greenhouse Structures

Two trials were conducted at two different sites and different time spans. The first site (site I) is located in the Kfarmashoun–Jbeil district, North of Beirut (34°07′25″ N 35°39′04″ E), at an altitude of 300 m, and the second site (site II) is located in Rmeileh, South of Beirut (33°36′30″ N 35°23′53″ E), at 100 m altitude. At each site, two commercial high plastic arched tunnels of 450 m^2^ (9 m × 50 m × 3.15 m/width × length × height) at site I, and 325 m^2^ (7 m × 46.5 m × 2.90 m) at site II, covered with 200 µm clear thermal polyethylene, were selected for the experiments. One tunnel served as pesticide control, where the farmer followed his normal pest control measures, and the second tunnel was used to implement the BIPM program. The entrance and vents were equipped with insect-proof nets.

### 4.3. Pre-Transplanting Measures in BIPM Greenhouses

In the BIPM tunnels, after soil preparation and a week before transplanting, yellow sticky cards and blue sticky cards were installed at a rate of 1 trap/16 m^2^ for attracting recently introduced or any whiteflies, thrips, and fungus gnats remaining from the previous crop. Blossomed marigold (Ferry-morse^®^, Norton, MA, USA) trap plants, one plant/pot, were placed in the BIPM tunnel at a rate of 1 flowering plant per 32 m^2^. Just before cucumber transplanting, marigolds were covered by a nylon bag and discarded to eliminate any insects that may have been attracted to the trap plants; new flowering marigolds replaced the removed ones. Weeds, inside and around the tunnels, were hand-pulled instead of applying herbicides and plant debris left from last season and were removed and burned. Data loggers (Ebro^®^, Ingolstadt, Germany) were installed to record daily temperature and relative humidity (RH), at intervals of 15 min. 

At site I (Kfarmashoun), the farmer reported that he had fumigated the soil with allyl isothiocyanates (Dominus^®^, Yuma, AZ, USA) in September, before the beginning of the fall growing season of cucumber and pepper. At the initiation of the experiment in March, the farmer requested to keep the pepper plants of the previous season (Fall) growing in the border rows. The pepper plants were pruned to reduce the overwintering stages of insects and mites. The two plastic tunnels were each equipped with 12 drip irrigation pipes. The two border rows contained 200 colored pepper plants of the “Tala^®^” variety remaining from the previous season, while in the ten middle rows 1000 cucumber seedlings, variety “Serena^®^”, were transplanted on March 6, at a density of 2.66 seedlings/m^2^; the growing period extended till mid-June. 

At site II (Rmeileh), the farmer reported conducting soil solarization in July/August. Each of the plastic tunnels was equipped with eight irrigation lines. A total of 900 seedlings (density 2.77/m^2^) of variety “Saifi^®^ (Robinson Agri, Mastita, Lebanon)” were transplanted on September 11. The growing period extended till the first week of December.

### 4.4. Post-Transplanting Measures

#### 4.4.1. Insect/Mite Scouting

A total of 50 randomly selected cucumber plants and 30 pepper plants were monitored per tunnel at weekly intervals. Three leaves (low, medium, and high)/plant were randomly scouted, totaling 150 cucumber leaves and 90 pepper leaves per tunnel at site I and 120 cucumber leaves at site II, ensuring that representative samples were collected from all areas within each tunnel. The number of arthropod pests and predators were counted: *T. urticae* nymphs and adults, *B. tabaci* instars and adults, *F. occidentalis* nymphs + adults, *P. presimilis*, and *A. swirskii*. During the season, nine hotspot releases of *P. presimilis* at a rate of 24 mites/m^2^ were conducted to control *T. urticae* (Table S2), and nine releases of *A. swirskii* at a release rate of 50 mites/m^2^ were conducted to control *B. tabaci* and *F. occidentalis* (Table S4). Throughout the growing period, highly infested marigolds and cucumber leaves/plants were removed from the BIPM tunnels, and new flowering marigolds were introduced. 

At site I, the farmer had to stop production in the control tunnel on June 21, two weeks earlier than planned, due to high infestation by TSSM, despite repeated sprays with acaricides. The data collected on temperature and relative humidity are provided in Figures S1 and S2.

At site II, four introductions of *A. swirskii* were carried out at a release rate of 25 mites/m^2^ at the beginning of the season (1st and 2nd releases) and increased with the growth of cucumber plants to 50 mites/m^2^ (Table S4). Three releases of *P. persimilis* were performed in hotspots due to the non-uniform spread of *T. urticae* populations. 

#### 4.4.2. Control Tunnels

At site I, the farmer spraying program included 14 pesticide sprays during the growing season, each consisting of a mixture of a minimum of three pesticide active ingredients, applied roughly at weekly intervals. Some applications contained up to four different pesticides in one spray. The main sprays were insecticides/acaricides targeting mites, thrips, and whiteflies, while the fungicides targeted mildews. Details of pesticides applied in the control tunnel, including application dates, active ingredients, and trade names, are provided in Table S1.

At site II, the farmer applied six sprays of pesticide mixtures in a manner similar to the farmer at site I, except that most sprays contained a mixture of two insecticides and one insecticide/acaricide without alternation between sprays (Table S3).

#### 4.4.3. Intrinsic Rate of Increase

The intrinsic rate of increase was calculated according to the following equation:

r = (N2 − N1)/(t2 − t1) 1/N1(1)

where r is the intrinsic rate of increase, N2 is the final population size, N1 is the initial population size, and t2 and t1 are the final and initial time, respectively [50].

### 4.5. Statistical Analysis

The collected data were analyzed using the statistical package for social sciences (SPSS 24 for Windows). Descriptive statistics were presented to summarize the study variables of interest as counts and percentages for the categorical variables and as means and standard deviations for the continuous ones. The normality of the variables was evaluated using the Kolmogorov–Smirnov and Shapiro–Wilk tests of normality. If both tests were statistically significant, indicating that the variables of interest follow a normal distribution, a two-way ANOVA (Analysis of Variance) was performed to assess the effects of the two variables (protection method, and time) and their interactions on the level of pest infestation. This was followed by testing for statistically significant differences between variables based on Bonferroni’s post hoc test used to address multiple comparisons. The presentation of sample means within a single replicate is pseudoreplication. All reported p-values were based on two-sided tests and were compared with a significance level of 5%. 

## 5. Conclusions

In conclusion, BIPM may represent a reliable environmentally friendly alternative to pesticides for food safety and for sustainable management of major arthropod pests of cucumber and pepper grown in high plastic tunnels, with an area below 500 m^2^ and relatively poor aeration, common in the Mediterranean region. The simultaneous use of *A. swirskii* and *P. persimilis* provided good control of three major pests: whiteflies, thrips, and TSSM. The local strains showed good adaptation to environmental conditions prevailing in these tunnels, and their local production may significantly reduce pest management costs. In addition to the reduction in pesticide use, other ecological advantages were observed at the end of the growing season. When the crop was removed, the predators spread to nearby open field crops, and fewer pests were present to migrate to other crops. Any BIPM approach should consider all potential pests that may infest the crop and not only the three major pests mentioned in this manuscript. The market availability of several selective pesticides, parasitic or predatory arthropods, and biopesticides may represent a good complement/supplement to the two local phytoseiid mites.

## Data Availability

The data presented in this study are available in the Supplementary Material.

## Supplementary Materials

The following supporting information can be downloaded at: https://www.mdpi.com/article/10.3390/plants13060889/s1, Figure S1: The average temperature at site I; Figure S2: The average temperature and Relative Humidity at site I; Figure S3: The average temperature and Relative Humidity at site II; Figure S4: Average temperature and Relative Humidity at site II; Table S1: Pesticide sprays in the Control greenhouse at site I; Table S2: Release dates and ratios of natural enemies in the BIPM greenhouse at site I; Table S3: Pesticide sprays in the Control greenhouse at site II; Table S4: Release dates and ratios of natural enemies in the BIPM greenhouse at site II.
