# Supplementary material for: Biological Control of Three Major Cucumber and Pepper Pests: Whiteflies, Thrips, and Spider Mites, in High Plastic Tunnels Using Two Local Phytoseiid Mites
Source: Plants (Basel). 2024 Mar 20;13(6):889. doi: 10.3390/plants13060889 (PMC10976136; doi:10.3390/plants13060889)
Supplement: Supplementary file 1 [file plants-13-00889-s001.zip › plants-2827135-supplementary.pdf]

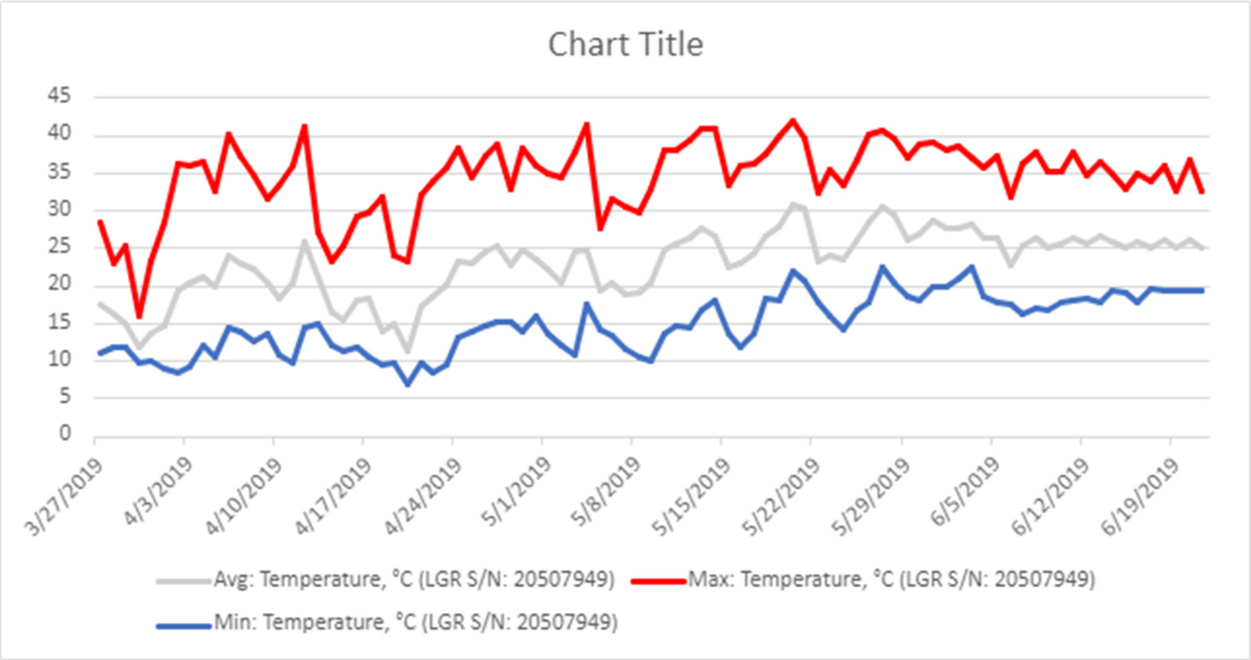

Figure S1. The average temperature at site I.

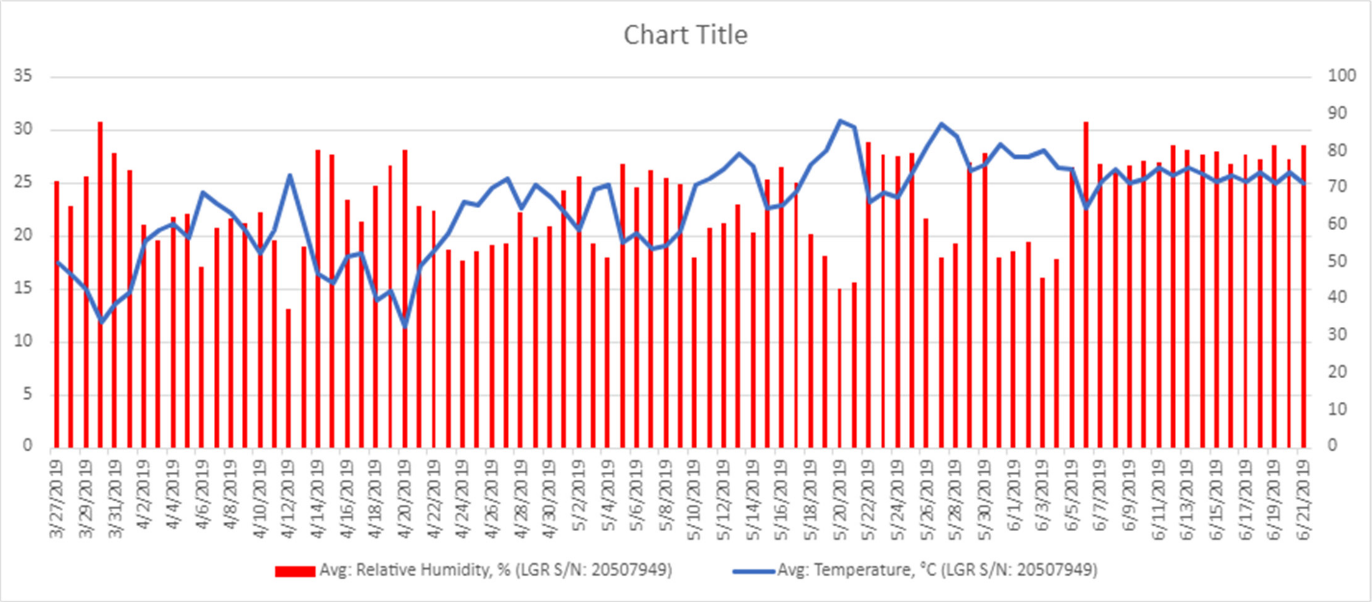

Figure S2. The average temperature and Relative Humidity at site I.

Table S1. Pesticide sprays in the Control greenhouse at site I.

Week	Date	Nb of sprays per week	Active Ingredient
0	27/3/2019	2	Emamectin Benzoate
			Acetamiprid
			Fenbutatin oxide
1	3/4/2019	1	Mancozeb + Cymoxanyl
			Acetamiprid
			BT
			Flonicamid
2	10/4/2019	1	Acetamiprid
			Tolfenpyrad
			Mancozeb
3	17/4/2019	0	-
4	24/4/ 2019	1	Chlorothalonil
5	1/5/2019	2	Abamectin
			Thiamethoxam
			Acetamiprid
			Tolfenpyrad
			Nemacic
6	8/5/2019	1	Thiamethoxam
			Acetamiprid
7	15/5/2019	1	Mancozeb
8	22/5/2019	1	Acetamiprid
			Abamectin
			Tolfenpyrad
			Carbosulfan
9	28/5/2019	0	-
10	3/6/2019	2	Acetamiprid
			Tolfenpyrad
			Thiamethoxam
			Mancozeb + Cymoxanyl

11	11/6/2019	1	Abamectin
			Acetamiprid
			Thiamethoxam
12	14/6/2019	0	-
12	18/6/2019	1	Abamectin
			Acetamiprid
			Thiamethoxam
			Pyridaben
14	21/6/2019	0	-

Table S2. Release dates and ratios of natural enemies in the BIPM greenhouse at site I.

Date	Week	*Amblyseius swirskii*	*Phytoseiulus persimilis*	*Beauveria bassiana*
March 27,2019	0	16,000	2,500	
April 3, 2019	1	8,000		
April 10 ,2019	2	27,000	1,200	10ˆ8 on hotspots
April 17 ,2019	3	-	-	-
April 24,2019	4	-	-	-
May 1,2019	5	15,000	-	10ˆ8 on hotspots
May 8,2019	6	26,000	700	10ˆ8 on hotspots
May 15,2019	7	10,000	1,000	
May 22,2019	8	5,000	1,000	
May 28,2019	9	-	5,000	
June 3,2019	10	-	500	
June 11,2019	11	-	2,000	

June 14,2019	12	-	2,000	
June 18,2019	13	-	-	

107,000/400= 267 15,900/4oo= 40/m2

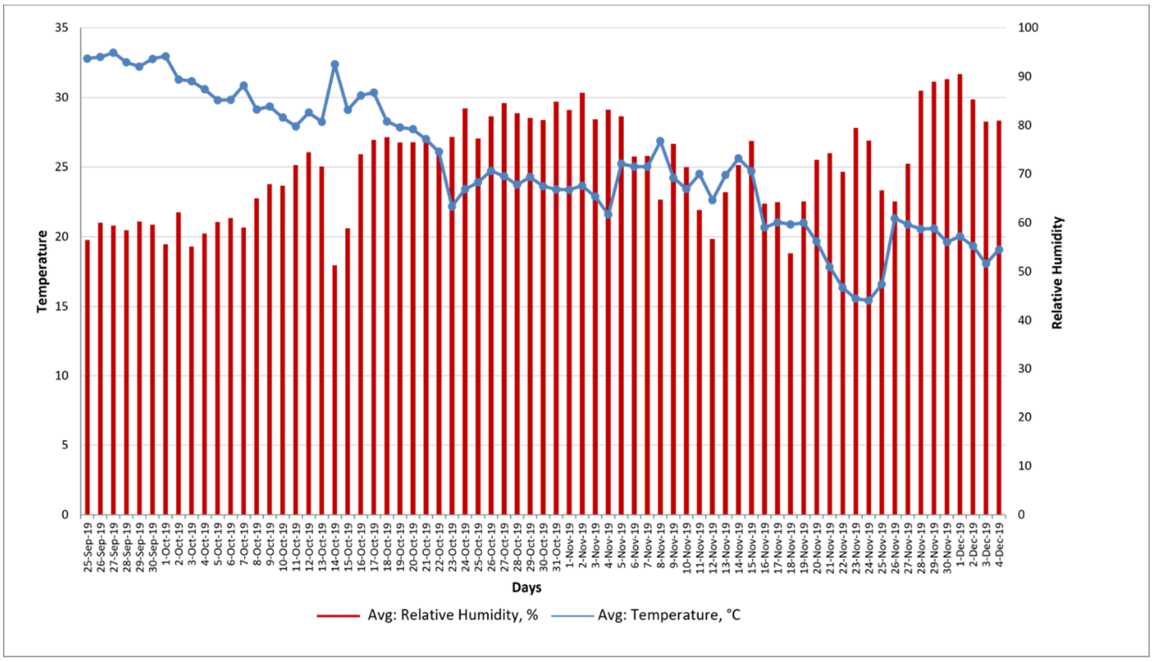

Figure S3. The average temperature and Relative Humidity at site II.

Table S3. Pesticide sprays in the Control greenhouse at site II

**Week**	**Date**	**Nb of sprays per week**	**Active Ingredient**
0	25/9/2019	1	Abamectin<br>Lambda- Cyhalothrin
1	2/10/2019	1	Acetamiprid (20%)
2	9/10/2019	1	Acetamiprid (20%)
3	16/10/2019	0	-
4	1/11/ 2019	1	Acetamiprid (20%)<br>Abamectin
5	6/11/2019	1	Acetamiprid (20%)<br>Abamectin
6	14/11/2019	0	-
7	20/11/2019	1	Abamectin<br>Lambda- Cyhalothrin
8	28/11/2019	0	-
9	4/12/2019	0	-

Table S4. Release dates and ratios of natural enemies in the BIPM greenhouse at site II

Date	Week	*Amblyseius swirskii*	*Phytoseiulus persimilis*	*Beauveria bassiana*
25/9/2019	0	8,125	-	-
2/10/2019	1	-	-	-

9/10/2019	2	8,125	1,000	-
16/10/2019	3	-	620	-
1/11/ 2019	4	16,250	1,500	10^8 spore/ml on hotspots
6/11/2019	-	-	-	10^8 spore/ml on hotspots
14/11/2019	6	16,250	-	10^8 spore/ml on hotspots
20/11/2019	7	-	-	-
28/11/2019	8	-	-	75L of 10^8 spore/ml)
4/12/2019	9	-	-	75L of 10^8 spore/ml)

48,750/325=150 3120/325=9.6

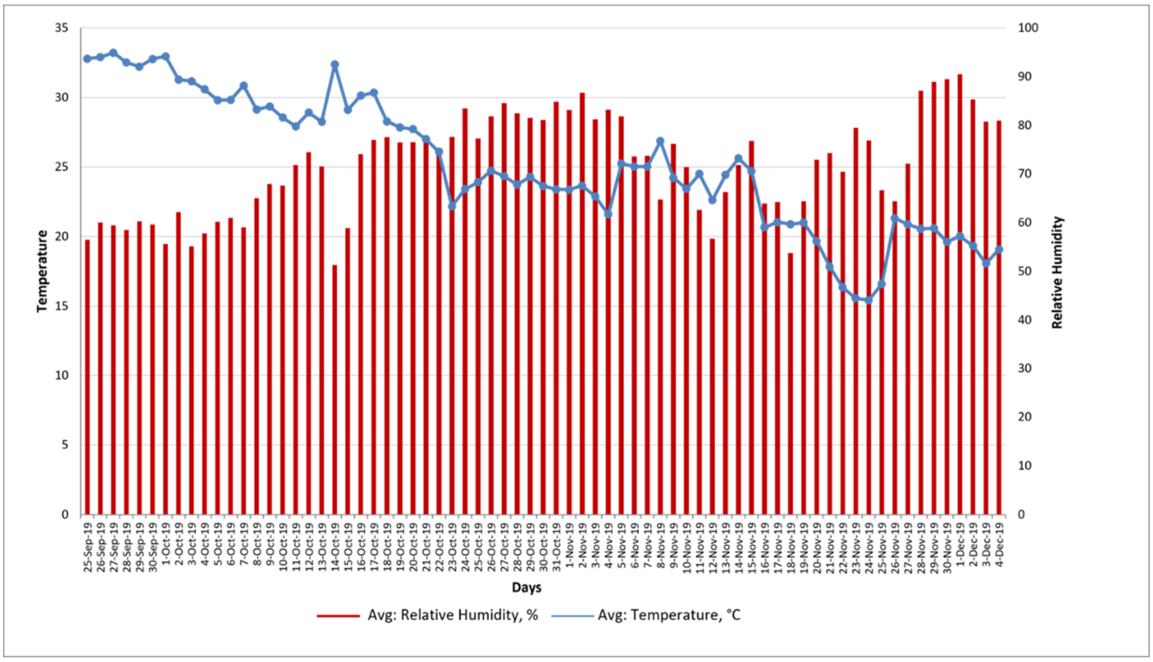

Figure S4. Average temperature and Relative Humidity at site II
